# Smoking cessation strategies for women: An analysis of smoking cessation determinants among Korean female smokers participating in a smoking cessation outreach program

**DOI:** 10.34172/hpp.2023.07

**Published:** 2023-04-30

**Authors:** Minhee Suh, Boae Im, Hun Jae Lee, Kyu-Sung Kim, Min Sohn

**Affiliations:** ^1^Department of Nursing, Inha University, 100 Inharo, Incheon, 22212, South Korea; ^2^College of Medicine, Inha University, 100 Inharo, Incheon, 22212, South Korea

**Keywords:** Women’s health services, Smokers, Smoking, Smoking cessation, Counseling

## Abstract

**Background:** Although there is strong evidence that behavioral counseling improves quit rates, limited data are available on individualized smoking cessation counseling provided to female smokers because they often are hesitant to identify as smokers. This study aimed to elucidate factors related to smoking cessation among Korean women who participated in the smoking cessation outreach program.

**Methods:** This retrospective descriptive study used data retrieved from the Korea Health Promotion Institute. The data included individual participant characteristics, supportive services received, and self-reported smoking cessation outcomes from June 1, 2015, to December 31, 2017.

**Results:** Data from 709 women were analyzed. We found cessation rates of 43.3% (confidence interval [CI]=0.40, 0.47) at four weeks, 28.6% (CI=0.25, 0.32) at 12 weeks, and 21.6% (CI=0.19, 0.25) at six months. Significant determinants of quitting at six months were regular exercise (odds ratio [OR]=3.02; 95% CI=1.28, 3.29; *P*=0.009) and the number of counseling sessions during the first four weeks of the program (OR=1.26; 95% CI=1.04, 1.82; *P*=0.041).

**Conclusion:** Providing intensive counseling during initial phase of smoking cessation program and regular exercise would be effective strategies for smoking cessation programs for women smokers to promote their health.

## Introduction

 Although the reported prevalence of smoking among Korean women was 4.4% in 2020, the sixth-lowest observed among Organization for Economic Cooperation and Development (OECD) member countries,^1^ the smoking rate among women is not decreasing. According to the national survey in Korea, the smoking rate among women increased from 5.5% to 7.5% for the last 3 years while that among men has declined steadily from 51.7% to 39.4% for the last decade.^2^ A research addressed there was no significant sex differences in tobacco smoking in adolescent,^3^ which implies further increase in smoking women. Furthermore, several researchers have reported that women who self-report as smokers comprise less than half of the cotinine-verified smokers in Asian countries,^4^ which implies a number of female smokers may be underreported.

 Recently, studies have evaluated the differential health effects of smoking, especially higher among women. They reported a significantly higher risk of cardiac disease and respiratory obstruction among women.^5,6^ Smoking is also associated with an increased risk of breast and cervical cancers in women.^7,8^ Moreover, female smokers report greater difficulty quitting smoking than men^9^ due to greater stress levels and hormonal fluctuations during the menstrual cycle.^10^

 However, it is difficult to identify female smokers and provide smoking cessation interventions because they often do not want to be recognized as smokers. As Meijer et al found that female smokers were more reluctant to positively rate conversations about smoking than male smokers,^11^ it is needed for clinicians to focus more on discussing smoking cessation with them. O’Keeffe et al also addressed the true risks of smoking on diseases among women may be underestimated due to lack of literatures.^12^ Therefore, it is not surprising that there are fewer studies on smoking cessation interventions for women than for men. It follows that promoting smoking cessation among female smokers is a public health priority.

 In 2015, the Korea Health Promotion Institute initiated the Outreach Program for Smoking Cessation, which targeted women who smoke. This program provides smoking cessation counseling and supportive assistance where smokers live and work. Although there is substantial evidence that behavioral counseling combined with pharmacotherapy improves quit rates,^13^ limited data on individualized smoking cessation counseling is available to female smokers. Since women are less likely to use pharmacotherapy,^14^ it is necessary to analyze the outcomes of smoking cessation programs. This includes counseling for successful smoking cessation and establishing effective strategies for creating a smoking cessation program for female smokers who are willing to quit but hesitate to seek help.

 Thus, this study examined the characteristics of female smokers enrolled in the outreach program for smoking cessation. The study assessed quit rates at four-week, twelve-week, and six-month intervals after enrollment to identify the determinants of successful smoking cessation among female smokers. Findings from this study will add to the knowledge base for establishing effective strategies to provide smoking cessation programs for female smokers.

## Material and Methods

###  Study design and participants

 This retrospective descriptive study used data from the Korea Health Promotion Institute. Participants were women enrolled in a smoking cessation outreach program at a regional smoking cessation center in South Korea from June 1, 2015, to December 31, 2017.

###  Outreach program for smoking cessation

 The program is a government-driven smoking cessation program developed by the Korea Health Promotion Institute and offered by regional smoking cessation centers. Women were recruited from residential communities, universities, and businesses with a higher proportion of female workers, such as telephonic customer services, small businesses, and factories. In order to find women smokers reluctant to participate in the smoking cessation program, business owners were persuaded to create an in-company atmosphere to encourage female workers to participate in the program and quit smoking for their health.

 The program provided a minimum of four in-person counseling sessions, nicotine replacement therapy (NRT) with nicotine patches and gums, and follow-up calls for six months. Counseling included discussions on individual barriers to smoking cessation, such as emotional or weight-related concerns, and building self-confidence. Counselors were registered nurses and psychological counselors trained in smoking cessation interventions and counseling. Smoking abstinence was evaluated by self-report at regular visits and follow-ups at four weeks, 12 weeks, and six months after enrollment. Counselors consulted a physician if participants required further pharmacotherapy. The counselors recorded all services in the Korea Health Promotion Institute’s electronic documentation system.

###  Data collection

 After the authors’ study proposal was approved by the institutional review board of their university (180608-1A) and the Central and Regional Smoking Cessation Center, they received the de-identified data from the Korea Health Promotion Institute (RTCC2018FH012). The data included participant characteristics, interventions, and smoking cessation outcomes. Counselors used structured interviews and anthropometric measurements at enrollment to collect original data on demographic and smoking-related characteristics.

 Demographic data included age, body mass index, education level, occupation, health insurance, regular exercise, and alcohol intake within the past year. Detailed information on smoking status and history was also taken. The data included quantity smoked, expired level of carbon monoxide (CO), nicotine dependence, craving times, age at which smoking began, years of smoking, and attempts to quit within the past year. Reasons for participating in the program, motivation, confidence, and readiness for smoking cessation were also recorded. Nicotine dependence was evaluated using the Fagerström Test for Nicotine Dependence (FTND). To measure motivation, confidence, and readiness for smoking cessation, the participants were asked to answer to the questions, “How important is it for you to quit smoking for good?”, “How confident are you of quitting smoking?”, “How ready are you to quit smoking?”, respectively.^15^ The participants’ responses were recorded on a 10-point Likert scale ranging from 0 (*not at all*) to 10 (*very much*) for each question.

 Data on the smoking cessation intervention and outcomes included frequency of counseling during the first four weeks to six months of the program, pharmacotherapy as a cessation aid, and self-reported smoking cessation rates at four weeks, 12 weeks, and six months.

###  Data analysis

 Data analysis was conducted using IBM SPSS Statistics 25 for Windows (IBM, Armonk, NY, USA). Demographic and smoking characteristics, intervention data, and outcomes were analyzed using descriptive statistics. Multivariate logistic regression was performed to differentiate between contributing factors and their impact on successful smoking cessation at four weeks, 12 weeks, and six months. Results were presented as odds ratios (ORs) and 95% confidence intervals (CIs). Multivariate logistic regression analysis included age and significant factors in the univariate analysis (*P*< 0.05). We used the Hosmer–Lemeshow goodness-of-fit statistic with the area under the receiver operating characteristic curve for testing model. The level of statistical significance was set at α < 0.05.

## Results

###  Demographic and smoking characteristics of the participants

 Data from 709 female participants were included in the analysis, and the average age of participants was 32 years old. The average body mass index (BMI) was 21 kg/m^2^, and 12.6% of participants engaged in regular exercise. The majority of the participants (93.1%) had graduated from high school, and 4.5% had graduated from college. Regarding occupation, 33.4%, 20.2% and 11.3% of participants were service workers, college students, and craft workers, respectively, while 5.2% were not employed. Of the 589 participants, 48.6% reported consuming alcohol on at least one occasion within the past year and 12.6% were on regular exercise.

 The smoking-related characteristics of the participants at enrollment are shown in Table 1. The participants smoked an average of 10.4 (SD: 6.5) cigarettes per day, and their expired CO level was 10.0 ± 8.3 on average. A total of 60.4% showed low nicotine dependence, 32.7% showed medium nicotine dependence, and 6.9% showed severe nicotine dependence. The average age of smoking initiation was 20.0 (SD: 7.1), and years of smoking was 21.6 (SD: 4.6) years. Approximately 32% of the participants had tried quitting within the past year. The primary reason for quitting/enrolling in the smoking cessation outreach program was being asked to quit by family and friends (46.8%).

**Table 1:** Smoking characteristics of participants on enrollment (n = 709)

**Characteristics**	**No. (%) or mean±SD**
Smoking amount per day (cigarette)	10.4 ± 6.5
Expired CO (ppm)	10.0 ± 8.3
Nicotine dependency	
Low	428 (60.4)
Medium	232 (32.7)
Severe	49 (6.9)
Timing of craving for smoking (multiple answers allowed)	
After meal	372(52.5)
Negative situation	224 (31.6)
Habitual	195 (27.5)
After awakening in the morning	140 (19.7)
Age you first smoked (years)	20.0 ± 7.1
Years of smoking (years)	21.6 ± 4.6
Attempt to quit within the last one year, yes	229 (32.3)
Primary reason for quitting in this program	
At the request of family and friends	332 (46.8)
My health	275 (38.8)
Price of cigarettes	55 (7.8)
A desire for better hygiene	25 (3.5)
Motivation for smoking cessation	7.0 ± 2.1
Confidence for smoking cessation	5.8 ± 2.0
Readiness for smoking cessation	5.8 ± 2.1
Number of smoking cessation counseling sessions in the first four weeks	4.6 ± 1.6
Number of smoking cessation counseling sessions for six months per person	7.7 ± 3.6
Use of medication therapy, yes	9 (1.3)
Rates of smoking cessation by self-report	
Four weeks	307 (43.3)
12 weeks	203 (28.6)
Six months	153 (21.6)

CO, carbon monoxide.

###  Interventions provided and rates of smoking cessation

 The average number of counseling sessions for smoking cessation during the first four weeks and the following six months were 4.6 (SD: 1.6) and 7.7 (SD: 3.6), respectively (Table 1). Only nine participants received further pharmacotherapy to help them quit. The smoking cessation rate was 43.3% at four weeks, 28.6% at 12 weeks, and 21.6% at six months.

###  Determinants of successful smoking cessation in logistic regression analysis 

 Logistic regression analysis was used to identify the determinants of successful smoking cessation among the participants at four weeks, 12 weeks, and six months (Table 2).

**Table 2:** Determinants of successful smoking cessation using logistic regression analysis (n = 709)

**Characteristics**	**4 Weeks**			**12 Weeks**			**4 Months**		
	**AOR**	***P***	**CI**	**AOR**	***P***	**CI**	**AOR**	***P***	**CI**
Age	0.98	0.430	0.95 -1.12	1.03	0.449	0.95-1.02	1.03	0.444	0.95-1.02
Education level									
< High school graduates	Ref								
High school graduates	0.41	0.152	0.10 -1.34	0.12	0.087	0.12-1.39	0.20	0.091	0.12-1.39
≥ College	0.71	0.651	0.14-4.50	0.25	0.349	0.16-3.16	0.56	0.616	0.16-3.17
Alcohol consumption within last one year, yes	1.95	0.039	1.07-2.68	0.78	0.529	0.32-1.46	0.61	0.185	0.32-1.45
Regular exercise, yes	1.77	0.069	0.21-3.54	3.22	0.019	1.96-3.26	3.02	0.009	1.28-3.29
Number of cigarettes/day	0.94	0.008	0.80-0.95	0.87	0.002	0.90-0.98	0.94	0.103	0.90-1.02
CO at baseline	0.97	0.056	0.97-1.07	1.02	0.493	0.95-1.01	0.98	0.489	0.95-1.01
Nicotine dependence									
Low	Ref								
Medium	1.09	0.704	0.48-2.44	1.08	0.849	0.67-1.76	0.92	0.840	0.67-1.76
Severe	2.11	0.099	0.23-5.02	14.86	0.005	2.87-5.14	4.24	0.054	0.87-5.14
Years of smoking	1.01	0.705	0.88-1.08	0.98	0.614	0.96-1.10	0.96	0.332	0.96-1.60
Attempt to quit within the last one year, yes	1.63	0.039	1.46-2.00	0.94	0.880	0.36-2.58	1.42	0.321	0.75-2.58
Motivation for smoking cessation	1.11	0.067	0.91-1.33	1.10	0.324	0.99-1.25	1.05	0.597	0.99-1.25
Confidence for smoking cessation	1.05	0.588	0.08-1.77	1.38	0.011	1.89-1.23	1.16	0.198	0.89-1.23
Readiness for smoking cessation	1.12	0.154	0.77-1.21	0.97	0.760	0.96-1.32	1.04	0.732	0.96-1.32
Number of counseling sessions during the first four weeks				1.60	0.001	1.20-2.14	1.26	0.041	1.04-1.82

AOR, adjusted odds ratio; CI, confidence interval; Ref, reference category; CO, expired carbon monoxide.

 At four weeks, participants who consumed alcohol at least once in the past year or who had tried to quit smoking within the past year were 1.95 times (*P*= 0.039) and 1.63 times (*P*= 0.039) more likely to successfully quit smoking, respectively. However, these effects were not observed at 12 weeks or six months. Numbers of cigarette per day were 0.94 times more likely to have quit successfully by four weeks (*P*= 0.008). The Hosmer–Lemeshow goodness of fit was not significant (χ^2^ = 3.625, *P*= 0.889), indicating good model fit. The ROC curve area of the model was 0.735.

 At 12 weeks, participants who exercised regularly were 3.22 times more likely to have quit successfully by four weeks (*P*= 0.019). Participants with severe nicotine dependence were 14.86 times more likely to quit than participants with low nicotine dependence (*P*= 0.005). Participants with higher confidence in their ability to quit smoking were 1.38 times more likely to quit at 12 weeks (*P*= 0.011). In contrast, neither motivation nor readiness for smoking cessation was a significant determinant of successful smoking cessation. Participants who had received more counseling during the first four weeks were 1.60 times more likely to quit at 12 weeks (*P*= 0.001). The Hosmer–Lemeshow goodness of fit was not significant (χ^2^ = 10.070, *P*= 0.260), indicating adequate model fit. The ROC curve area of the model was 0.804.

 At six months, participants who exercised regularly and who received more counseling during the first four weeks were 3.02 (*P*= 0.009) and 1.26 (*P*= 0.041) times more likely to quit, respectively. The Hosmer–Lemeshow goodness of fit was not significant (χ^2^ = 3.194, *P*= 0.922), indicating good model fit. The ROC curve area of the model was 0.760.

## Discussion

 In our study, the majority of the participants enrolled in the smoking cessation program were high school graduates and not college graduates. This is inconsistent with previous findings that the majority of female smokers participating in smoking cessation programs were college graduates.^16^ In previous studies, female smokers who had not completed college were shown to have a lower intention to quit smoking.^17^ Therefore, it is encouraging that the participants in this study had indicated an intention to quit smoking and participate in the smoking cessation program despite a lower level of education. There have been extensive campaigns in South Korea targeting less-educated female smokers to increase the awareness of the need to quit smoking and participate in smoking cessation programs. This may be why our participants had been highly motivated to participate in smoking cessation program, even though 67.7% of them had not tried to quit before. On the other hand, participants in our study reported wanting to quit smoking for the benefit of family and friends, which is consistent with findings that guilt due to exposing children to passive smoke inhalation worked as a powerful motivator to quit among female smokers.^18^ These characteristics should be considered when designing strategies to improve the smoking cessation rate for female smokers.

 The smoking cessation rates in our study were 43.3%, 28.6%, and 21.6% at four weeks, 12 weeks, and six months, respectively. The quit rates were relatively high compared to previously reported rates of 36% at four weeks in female smokers^19^ and 12.2% at six months in female smokers.^20^ The previous study^20^ provided a smoking cessation program that included telephonic counseling and supportive ambassadors. Our program focused on in-person counseling alongside NRT, which could be effective for promoting smoking cessation among female smokers. A recent study reported a six-month quit rate of 24.1% of female smokers after providing them with 26-weeks of personalized NRT and supportive counseling,^21^ which is similar to our findings. Women have unique barriers to quitting such as post-cessation weight gain,^22^ menstrual cycle effects,^23^ and a lower likelihood of utilizing smoking cessation services.^14^ It follows that tailored smoking cessation counseling based on in-person meetings is an important factor for female smokers to quit smoking.

 Nevertheless, frequent in-person counseling may not always be possible due to limited resources. To find the most efficient way to provide counseling, we need to focus on our finding that frequent counseling sessions during the first four weeks were associated with smoking cessation at 12 weeks and at six months. This would be an effective strategy for women because the initial phase of smoking cessation is more challenging for females than for male smokers.^24^ Myers et al also pointed out the importance of the initial treatment phase of smoking cessation.^25^ Frequent counseling in the initial period of the program helped our female participants to maintain smoking cessation throughout the follow-up period up to six months in our study. Therefore, counseling in the initial period is expected to positively affect the retention rate­—a strong predictor of smoking cessation.^26^ Consequently, it seems an effective strategy to provide more counseling sessions in the early period of the program for women.

 In our study, participants who exercised regularly were three times more likely to abstain from smoking at 12 weeks and six months, contrary to a previous Cochrane review study which insisted that exercise did not improve long-term abstinence.^27^ Martin-Sanchez et al also noted that exercise can alleviate negative mood, depression, and post-cessation weight gain among female smokers.^28^ Issues such as negative mood and weight gain are considered important smoking-associated concerns, especially in female smokers^22,29^; using exercise to alleviate these issues is still considered as an effective strategy. Although further studies are needed to prove the benefits of exercise on smoking cessation among female smokers, regular exercise is likely to help female smokers quit smoking to promote their health.

 Interestingly, participants with severe nicotine dependence were much more likely to quit, although the number of cigarettes per day was negatively associated with quitting in our study. Contrary to our results, Kim and Lee showed that severe nicotine dependence was a factor in smoking cessation failure in female college students.^30^ There are differences, however, in the ages of the participants. The majority of our participants were female smokers older than 20, whereas those in the study by Kim and Lee were all college students with average age of 20 (SD: 2.63). Because older female smokers are more concerned about their health than younger female smokers, severe nicotine dependency might have been a facilitating factor for smoking cessation. Second, the FTND might be a less reliable measure of nicotine dependence among Korean female smokers due to sociocultural factors. They were more likely to smoke at home or in bed because of their reluctance to smoke in public places, which may lead to higher FTND scores, exaggerating their nicotine dependence. Indeed, we found a weak correlation between the number of cigarettes per day and the total FTND scores in our participants. Kim and colleagues have also reported different psychometric properties of the FTND among Korean American female smokers.^31^

 In our study, motivation levels and readiness for smoking cessation were not found to be powerful determinants of successful smoking cessation at 12 weeks. Rather, confidence in smoking cessation had a significant influence on smoking cessation at 12 weeks (but it was not significant at six months). This is contrary to previous findings that suggested an association between successful smoking cessation and motivation to improve health among men.^32^ It seems that our participants were already motivated and ready to quit because of extensive smoking cessation campaigns targeting marginalized smoking populations, such as women in South Korea. A previous study also found that motivation and readiness only affected attempts to quit, but not the three-month abstinence from smoking.^33^ The findings suggest that counseling strategies to promote confidence would be effective in retaining female smokers on the program.

 Our study had several limitations. First, our quit rate could be overestimated because we evaluated quitting through self-reporting, and the dataset had a minimal amount of CO or cotinine verification. Second, potential confounding variables, such as mental health problems and the use of different tobacco products, were not measured. Third, our findings cannot be generalized because factors associated with quit rate differ across social, cultural, and economic environments.^34^ Nevertheless, our study is significant because it focused on smoking cessation in a large number of Asian female smokers while most smoking cessation research literature has focused on non-Asian smokers. Further studies are needed to investigate the connection between female smokers’ mental health and smoking cessation alongside biological verification, and to increase initial counseling sessions for them to promote quit rate.

## Conclusion

 In summary, smoking cessation rates of 43.3% at four weeks, 28.6% at 12 weeks, and 21.6% at six months in our Korean female smokers. Significant determinants of quitting at six months were regular exercise and the number of counseling sessions during the first four weeks of the program. Smoking cessation management policies should be developed focusing on improvement of initial counseling sessions with the emphasis of regular exercise for long-term smoking cessation in Korean female smokers.

## Acknowledgements

 This study used data from the Korea Health Promotion Institute. The authors are solely responsible for the study’s results.

## Competing Interests

 The author(s) declare no potential conflicts of interest concerning the research, authorship, or publication of this article.

## Ethical Approval

 This study was approved by the institutional review board of Inha university (180608-1A) and the Central and Regional Smoking Cessation Center.

## Funding

 The study was supported by the Health Promotion Fund, Ministry of Health & Welfare, Republic of Korea (RTCC2018FH012).
